# Research on Field Soybean Weed Identification Based on an Improved UNet Model Combined With a Channel Attention Mechanism

**DOI:** 10.3389/fpls.2022.890051

**Published:** 2022-06-15

**Authors:** Helong Yu, Zhibo Men, Chunguang Bi, Huanjun Liu

**Affiliations:** ^1^College of Information Technology, Jilin Agricultural University, Changchun, China; ^2^Northeast Institute of Geography and Agroecology, Chinese Academy of Sciences, Changchun, China

**Keywords:** semantic segmentation, weed recognition, feature fusion, channel attention mechanism, improved UNet model

## Abstract

Aiming at the problem that it is difficult to identify two types of weeds, grass weeds and broadleaf weeds, in complex field environments, this paper proposes a semantic segmentation method with an improved UNet structure and an embedded channel attention mechanism SE module. First, to eliminate the semantic gap between low-dimensional semantic features and high-dimensional semantic features, the UNet model structure is modified according to the characteristics of different types of weeds, and the feature maps after the first five down sampling tasks are restored to the same original image through the deconvolution layer. Hence, the final feature map used for prediction is obtained by the fusion of the upsampling feature map and the feature maps containing more low-dimensional semantic information in the first five layers. In addition, ResNet34 is used as the backbone network, and the channel attention mechanism SE module is embedded to improve useful features. The channel weight is determined, noise is suppressed, soybean and grass weeds are identified, and broadleaf weeds are extracted through digital image morphological processing, and segmented images of soybean plants, grass weeds and broadleaf weeds are generated. Moreover, compared with the standard semantic segmentation models, FCN, UNet, and SegNet, the experimental results show that the overall performance of the model in this paper is the best. The average intersection ratio and average pixel recognition rate in a complex field environment are 0.9282 and 96.11%, respectively. On the basis of weed classification, the identified weeds are further refined into two types of weeds to provide technical support for intelligent precision variable weed spraying.

## Introduction

Weeds are one of the main reasons for crop yield and quality decline (Hamuda et al., 2016). They compete with crops in a field (such as soybeans) for sunlight, water, nutrients, and living space (Hongbo et al., 2020). At present, the main cleaning and control method for weeds is to spray herbicides on a large area (Ma et al., 2011), but this method not only causes much pesticide waste but also damages the ecological environment (Rodrigo et al., 2014), affects the quality of crops, and threatens human health (Yue et al., 2016). Weeds can be divided into two types, grass weeds and broadleaf weeds, according to the shape of their leaves (Javier Herrera et al., 2014). If different herbicides are applied to specific types of weeds, the weed control effect will be better. Grass weeds are monocotyledonous plants, their embryos have one cotyledon, their leaves are usually narrow and long, they have parallel veins, they do not have petioles, their leaf sheaths are open, and they have ligules (Li et al., 2015). Broadleaf weeds are also called dicotyledonous weeds. Their embryos have two cotyledons, they are herbaceous or woody, and they have reticulated veins and wide leaves (Hong-jun et al., 2009). Therefore, the rapid and accurate identification of different types of weeds is very important for the subsequent precise variable weed spraying. At present, machine vision recognition has been widely used in weed identification because of its advantages of fast and easy operation and noncontact and nondestructive target detection abilities (Wang et al., 2019). Machine vision recognition methods are mainly based on the different characteristic information of weeds and crops in images, such as color (Wenhua et al., 2009; Han et al., 2016), shape (Li et al., 2010), and texture (Hongyan and Jixing, 2014; Bakhshipour et al., 2017), to realize the identification of weeds and crops. Based on traditional machine learning algorithms, due to the limitations that arise when using a single feature in the process of weed identification, and to further improve the robustness of identification, it is necessary to fuse different features for identification (Ahmed et al., 2012; He et al., 2013; Zhao and Wei, 2014; Wang and Li, 2016). Although the above research has achieved certain results in weed identification, these methods rely on the manual selection of features and can only identify weeds satisfactorily under specific circumstances; they cannot identify weeds in complex and changeable field environments. Efficient extraction methods are less practical in addressing the problem of weed identification. In recent years, with the development of deep learning, convolutional neural networks have been used to automatically extract the deep features of weeds from images because they do not need to rely on a designer’s experience to select features, which has become a research hotspot. There are two main methods for target recognition after extracting image features based on deep convolutional neural networks. One is to draw a rectangular detection frame around a recognized target, and the other is to perform pixel-level classification on a recognized target (Asad and Bais, 2019). In the target detection method of drawing a rectangular detection frame around a recognition target (Peng et al., 2019; Meng et al., 2020; Jin et al., 2021), since the detection frame also contains a large area of background in addition to the target weeds, it is impossible to accurately distinguish the two types of weeds in the image, which affects the subsequent precise variable weed spraying. In view of the above problems, this paper uses semantic segmentation to study weed identification. Semantic segmentation predicts the category of each pixel in an image according to the prelabeled category (Jiaxing and Yujie, 2021) with an improved UNet structure and embeds the channel attention squeeze and excitation (SE) module to perform pixel-level segmentation on grass weeds and soybeans in an image. After identifying the grassy weeds and soybean pixel categories, other green pixels in the image are extracted by the digital image morphological processing method to extract broadleaf weeds to achieve the goal of simultaneously identifying two types of weeds, which is the next step. Intelligent equipment provides a reference for weed identification in variable pesticide spraying.

## Test Data and Preprocessing

### Image Acquisition

The soybean and weed image data used in the experiment were collected from the soybean experimental field of Jilin Agricultural University. Through manual intervention at an early stage, the images collected showed the growth status of soybeans; the dataset included images with soybeans alone, soybeans and grassy weeds, and soybeans and broadleaf weeds. Leafy weeds are associated with soybeans, grasses, and broadleaf weeds. The collection time was in mid-June 2021, and the time period was from 9:00 to 15:00. In order to ensure that the weed dataset can fully reflect the complexity of the natural environment, we selected different weather conditions such as sunny and cloudy days for image acquisition. The device used was a Huawei mate30 mobile phone positioned vertical to the ground at 60 cm above the ground, and the resolution was 3,000 × 4,000 pixels. The images are in JPG format.

### Image Preprocessing

**Figure 1:**
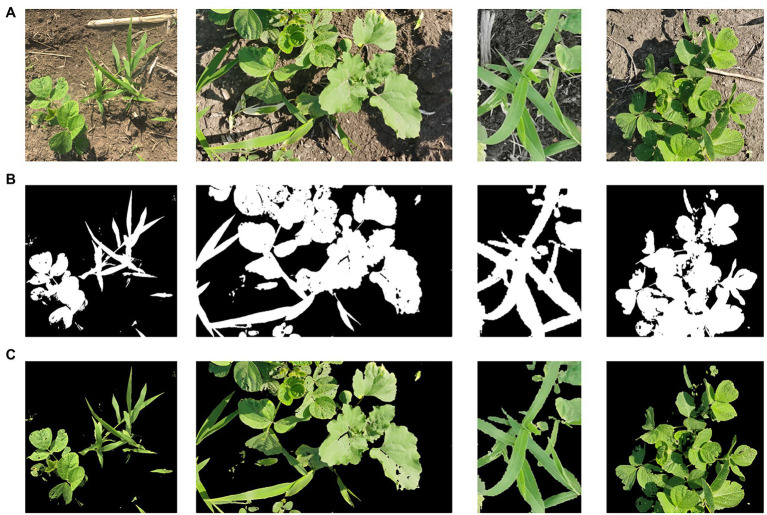
Hyper green feature algorithm **(A)** RGB original image, **(B)** Binary image, and **(C)** Final image.

The weed data of the object of this study were collected from a natural environment in a field. The background of a collected image mainly includes soil, stones, straw, and plant residue. To suppress the complexity of the field environment, the identification target and the background are segmented, and only the plants in the image are extracted. The part of interest is retained, thereby increasing the accuracy and efficiency of the weed identification model. The ultragreen feature algorithm (2 g-rb) is selected to increase the weight of the green channel in the image, improve the contrast with the nongreen background part, and extract the green crop and weed information (Zhao et al., 2009). First, 2 g-rb is used to obtain the grayscale image and histogram, and then the Otsu method (OTSU) is used to binarize the grayscale image of 2 g-rb to obtain a binary image. This is used as a mask to obtain a green crop image, as shown in Figure 1.

### Dataset Construction

**Figure 2:**
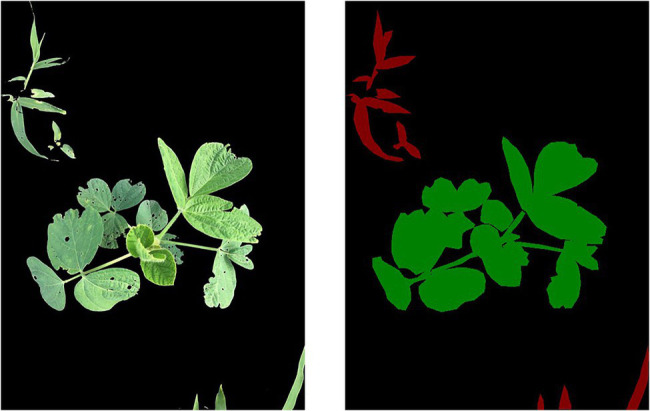
Original image annotation.

To enrich the training set of image data, better extract the features of the image, and prevent the built model from overfitting, data enhancement was performed on the 700 images collected in this experiment through operations such as rotations and color transformations. After data enhancement, the number of samples is expanded by five times for a total of 4,200 images. A total of 3,600 images were randomly selected for the training set, 400 images were selected for the validation set, and 200 images were selected for the test set. The labelme tool was used to manually label the image and label the identified objects, such as soybeans and grass weeds, as shown in Figure 2. The background (black) has an RGB value of [0,0,0], soybeans (green) have an RGB value of [0,128,0], and grass weeds (red) have an RGB value of [128,0,0].

## Semantic Segmentation Model Construction

### Original UNet Model

**Figure 3:**
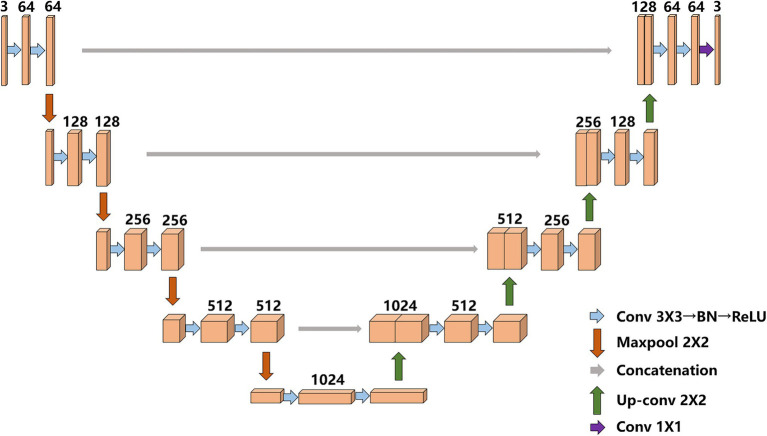
Original UNet model structure.

UNet was proposed by Ronneberger et al. (2015) at the 2015 MICCAI conference; it consists of an encoder (downsampling)-decoder (upsampling) structure, and the encoder stage consists of two 3 × 3 convolutional layers, a 2 × 2 maximum pooling layer is formed, and the activation function is a rectified linear unit (ReLU) function. A total of four downsampling operations are performed. After each pooling operation, the size of the feature map is reduced to half of the original size, and the number of channels is doubled. The decoder stage and the encoder part correspond to a total of four upsampling iterations through a 2 × 2 deconvolution layer (a transposed matrix). Each time the size of the upsampling feature map is doubled, the number of channels is halved. Different from other semantic segmentation networks, UNet combines the feature map obtained in the encoding stage with the feature map obtained in the decoding stage through skip connections to form a thicker feature map. UNet uses skip connections in the same stage. The final features are fused with more shallow features to retain more detailed information and make the segmentation results more refined. The traditional UNet model structure is shown in Figure 3.

### UNet Model Improvement

**Figure 4:**
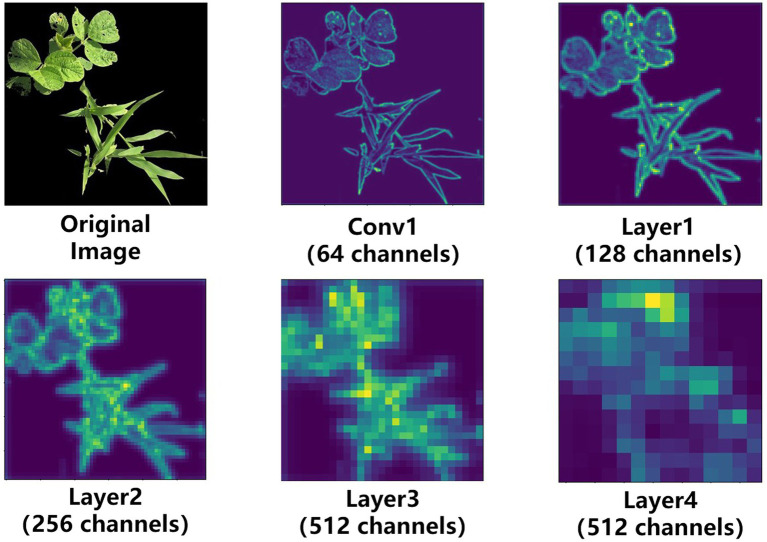
Feature visualization.

The visualization of shallow and deep feature maps is achieved through convolutional neural networks, as shown in Figure 4. We selected Resnet34 to extract the feature map of weeds and realize visualization. Some feature maps selected from different layers of visualization can summarize a little rule. The shallow network extracts texture and detail features, and the deep network extracts features such as contour and shape. Shallow networks contain more features and are also capable of extracting key features, such as the elongated shape of grass weeds. Relatively speaking, the deeper the number of layers, the more representative the extracted features, and as the number of layers increases, the resolution of the image becomes smaller and smaller. Since the soybeans and weeds to be segmented are similar in color, the main difference is in their shape and leaf area. The low-dimensional semantic information contained in the shallow feature map obtained in the encoding stage is the same as the high-dimensional semantic information contained in the deep feature map obtained in the decoding stage. There is a semantic gap when information is combined through skip connections. The final feature map used for prediction will lose some low-dimensional feature information, and the loss of these low-dimensional feature information will affect the segmentation accuracy of the weed boundary.

**Figure 5:**
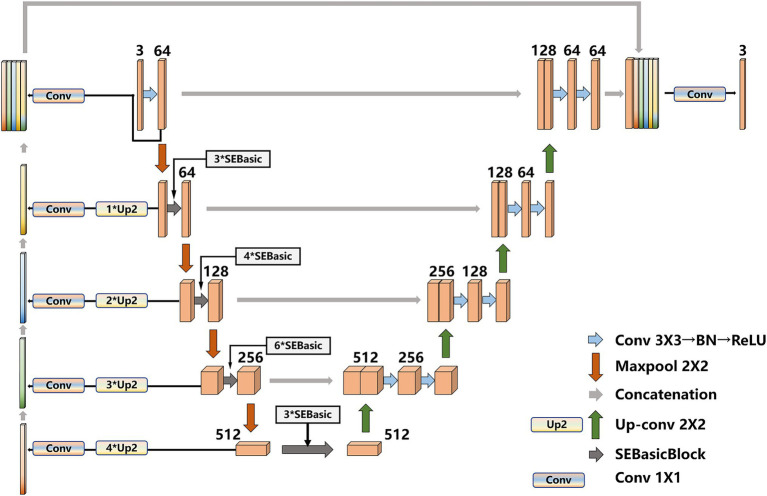
Improved UNet.

To eliminate the semantic gap, after the original image feature map is extracted by UNet network, our model builds a shallow feature fusion structure. After downsampling of original image, the feature map is upsampled by a 2 × 2 deconvolution layer (transposed matrix) for eight/six/four/two times, and the size of feature maps are restored to the original size, the feature maps are operated by Conv1 × 1, respectively, to realize cross-channel information interaction and integration, and reduce the latitude of the number of channels in the feature map. Then, the obtained feature map is concat connected with the final feature map. Finally, the feature map used for prediction is upsampled, and the contain features with more low-dimensional information. The shallow layers of feature maps are used because they contain more low-dimensional semantic information, such as shape and texture. The multilayer feature maps are combined because they have different receptive fields and can adapt to targets in different areas. The improved UNet model is shown in Figure 5.

### Backbone Network

**Figure 6:**
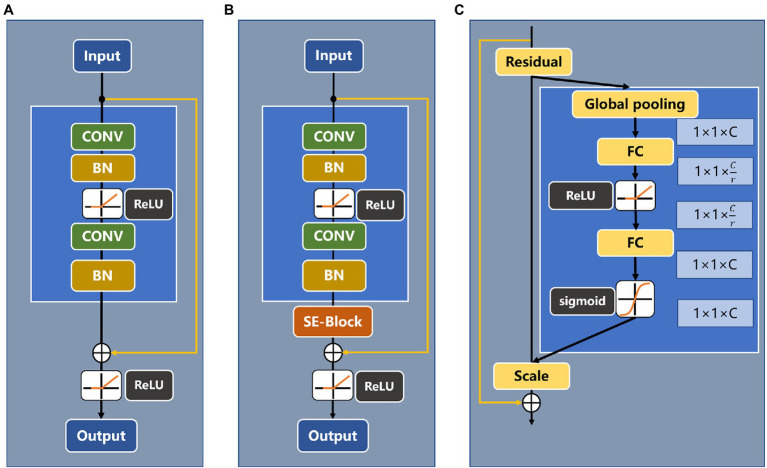
**(A)** Basic block of ResNet34, **(B)** Residual block of ResNet34 with an attention mechanism, and **(C)** SE-ResNet module.

Convolutional neural networks (Ronneberger et al., 2015; Jingjing et al., 2020; Zhang et al., 2020) have been widely used in image classification, target detection, semantic segmentation, and other tasks. The depth of a convolutional neural network has a great influence on subsequent recognition and classification. However, as the network depth is gradually increased, the phenomenon of gradient disappearance becomes increasingly obvious, and the network training performance appears to degrade. To solve the above problems, this paper adopts a ResNet (He et al., 2016) model as the backbone network to extract image features and introduces the residual block structure. Compared with ResNet18, ResNet34 is a deeper layer network and can learn more about identifying target features; it also has fewer parameters than ResNet50. Therefore, this paper selects ResNet34 as the backbone extraction network. Figure 6 shows the basic residual block structure of ResNet34 and the embedded SE module.

### Attention Mechanisms

**Figure 7:**
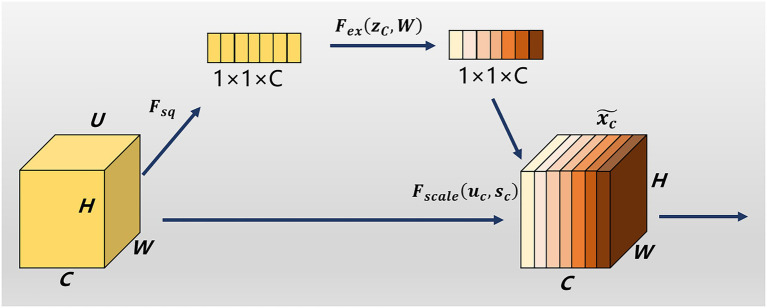
A squeeze-and-excitation block.

Since the data in this paper are collected in a complex natural field environment, there is considerable noise in the feature extraction process. To overcome the propagation of noise information in the feature channel, the attention mechanism SE module is introduced into the residual module (Hu et al., 2018). The SE module can explicitly model the interdependence between feature channels, thereby adjusting the weight of each channel; suppressing the weights of feature channels that are not related to the recognition target, such as noise channels; and enhancing the useful feature weights. A schematic diagram of the SE module structure is shown in Figure 7 and includes a squeeze module (squeeze) and an excitation module (excitation).

First, through the squeeze operation, the feature map U is input, the size is W × H, the number of channels is C, the average pooling algorithm is selected so that it has a global receptive field, and the input of W × H × C is generated as 1. The channel descriptor of ×1 × C is used for the output, and the extrusion formula is


(1) $$Z_{C}=F_{sq}\left(U_{c}\right)=\frac{1}{W\times H}\overset{W}{\underset{i=1}{\sum }}\overset{H}{\underset{j=1}{\sum }}U_{c}\left(i,j\right)$$

where $Z_{C}$ is the output feature map, F_sq is the extrusion function, and $U_{c}$ is the input feature map. Then, the excitation (excitation) operation is performed, and the formula is


(2) $$s=F_{ex}\left(z_{C},W\right)=\sigma \left(g\left(z,W\right)\right)=\sigma \left(W_{2}\delta \left(W_{1}z_{C}\right)\right)$$

where *S* is the channel weight parameter, $F_{ex}$ is the excitation function, σ is the ReLU activation function, and δ is the sigmoid activation function. First, through a fully connected layer operation, $W_{1}\in R^{\frac{c}{r}\times c},$ where *r* is a compression parameter, which is used to reduce the number of channels, the number of parameters, and the complexity of the model. Then, the ReLU activation function for nonlinearization. Then, through a fully connected layer operation, $W_{2}\in R^{c\times \frac{c}{r}},$ the number of channels is restored. Finally, the channel weight coefficient is calculated by the sigmoid activation function and multiplied by the original feature to realize the recalibration of the feature. The formula is


(3) $$\tilde{x_{c}}=F_{scale}\left(u_{c},s_{c}\right)=s_{c}u_{c}$$

where $\tilde{x_{c}}$ is the adjusted C-th channel feature, and $F_{scale}$ is the product function of the C-th channel weight parameter $s_{c}$ and the input feature map $u_{c}.$

### Test Platform

The test platform is equipped with an Intel® Core™ i7-9800X CPU clocked at 3.8 GHz, with eight cores, 16 threads, 64 GB memory, and an NVIDIA RTX2080Ti GPU with 11 GB video memory. A Windows 10 operating system, the Python programming language, a Pycharm-integrated development environment, and a PyTorch deep learning framework are applied.

#### Data Augmentation During Training

**Figure 8:**
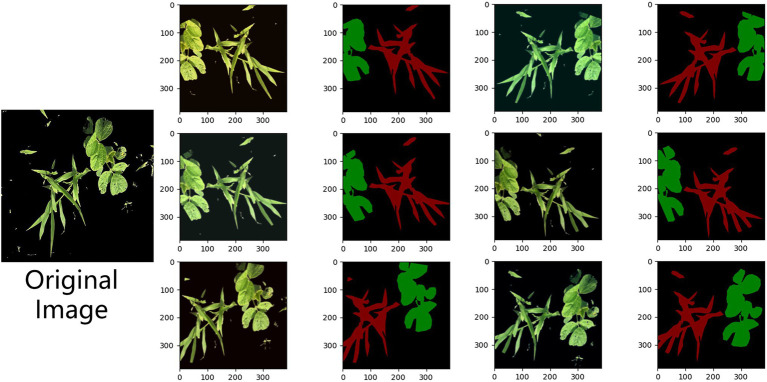
Data augmentation during training.

Due to the limited number of training samples, dataset enhancement is also required during training. During training, the original image and label are randomly cropped, rotated, blurred, and randomly moved by the RGB channel, as shown in the figure, and the obtained image is adjusted to the network input size of 512 × 512 pixels and sent to the network for training. It can adapt to images and recognition targets of different scale transformations, which is due to the increases generalization ability of the network model (Figure 8).

#### Model Training

Deep learning model training requires a large amount of sample data to make the model perform better. The amount of weed data collected in this paper is small, and it takes considerable time and effort to label the data. To improve the generalization ability of the network, the transfer learning method is adopted. To train the model, the backbone network trains the pretrained ResNet34 model on the ImageNet dataset as an initialization parameter. The number of model training epochs is 500 epochs, and the batch training size is 8. The learning rate is dynamically adjusted. The initial learning rate is 0.01. As the number of iterations increases, the learning rate gradually decreases, with a minimum of 0.001. The momentum factor is 0.9, the Adam optimization algorithm is used, and the cross-entropy loss function is used.

#### Evaluation Indicators

To prove the effectiveness of the semantic segmentation method, it is necessary to strictly evaluate the semantic segmentation method. The evaluation indicators used in this paper mainly include the mean pixel accuracy (mPA) and the mean intersection over union (mIoU).

The mPA metric refers to the average pixel accuracy of each category, and the calculation formula is


(4) $$mPA=\frac{1}{3}\left(\frac{TP}{TP+FP}+\frac{TN}{TN+FN}\right)$$

The mIoU ratio refers to the ratio of the intersection and union of the numbers of pixels in the predicted area and the real area. mIoU is the most commonly used metric due to its simplicity and strong representation and is used by most researchers. to report results. The calculation formula is


(5) $$mIoU=\frac{1}{3}\left(\frac{TP}{TP+FP+FN}+\frac{TN}{TN+FN+FP}\right)$$

where true positive (TP) represents a true example; that is, in the prediction of category *i*, TP is the number of pixels predicted as category *i*. False positive (FP) denotes the number of pixels that are mispredicted as background or the number of pixels of other classes predicted as class *i*. True negative (TN) denotes the number of pixels that are correctly predicted as background or other category pixels. False negative (FN) denotes pixels that are mispredicted as class *i*, background, or other class pixels.

#### Broadleaf Weed Extraction

**Figure 9:**
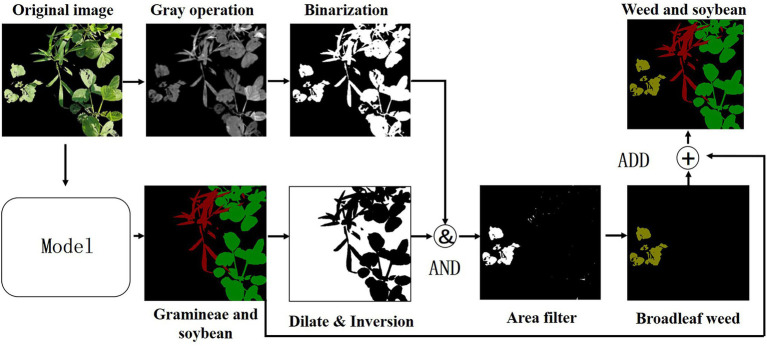
Broadleaf weed extraction process.

Based on the improved UNet model, to realize the identification of soybean and grass weeds, this paper extracts broadleaf weeds through a digital image morphological processing method (Wang et al., 2021). First, the grayscale image obtained by the 2 g-rb algorithm is used, and then OTSU is used to binarize the grayscale image of 2 g-rb to obtain a binary image. Then, the semantic segmentation model is used to identify the soybeans. After the expansion and inversion of the grassy weed prediction map, the binary image obtained from the original image is added to delete the grassy weeds and soybean parts predicted by the semantic segmentation model, and the remainder are broadleaf weeds. Moreover, the connected domain area filtering operation is performed on the obtained broadleaf weeds to remove small-area connected domains to prevent noise interference, and the broadleaf weeds are marked as (yellow) RGB = [128,128,0]. Finally, the obtained broadleaf weeds, the grass weeds, and the soybeans predicted by semantic segmentation are added to generate the final weed prediction image. The flowchart is shown in Figure 9.

## Results and Analysis

**Figure 10:**
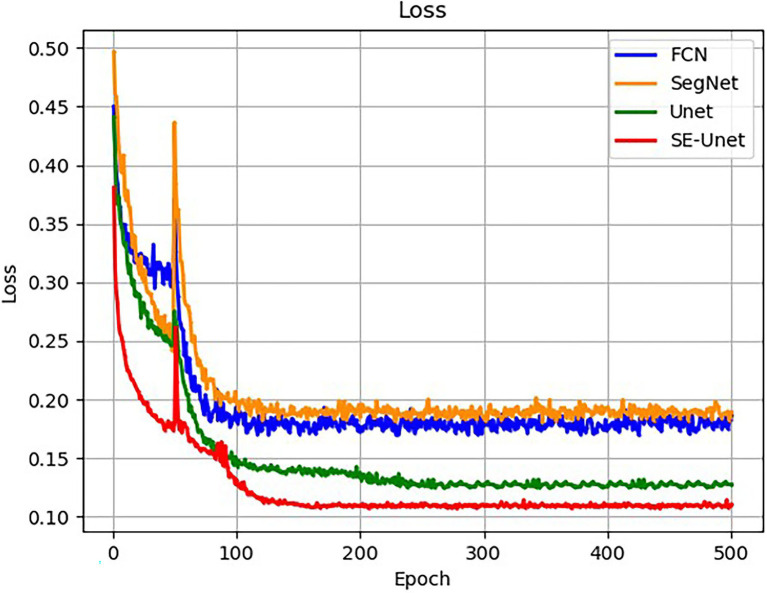
Training loss of the different models.

Under the same test platform conditions, the FCN, SegNet, UNet, and improved UNet semantic segmentation models were established, and the weed training set was used for training and testing. The loss function change curve of each network architecture is shown in the figure. The training is divided into two stages, namely, the freezing stage and the unfreezing stage. The first 50 epochs freeze the training parameters, and the feature extraction network does not change and occupies a small amount of video memory. Only network fine-tuning is performed. The last 450 epochs unfreeze the training parameters. At this time, the backbone of the model is not frozen, the memory occupied is large, and all the parameters of the network are changed. It can be seen from the figure that each model can achieve a good training process. The changes in the loss function values are basically the same. The loss decreases rapidly in the early stage of training, decreases steadily and slightly in the middle stage, and basically tends to stability in the later stage, and the network converges. At the final iteration, the improved UNet has the smallest loss value of 0.109 (Figure 10).

Table 1 shows that the intersection-over-union (IoU) value of the background is the largest among all categories, and the IoU values of soybeans are higher than those of grassy weeds.

**Table 1:** Performance comparison of the different models.

Model	IoU			mIoU	mPA%
	Grass	Soybean	Background		
FCN	68.31	84.58	88.67	80.52	88.18
SegNet	70.34	91.19	92.28	84.61	91.76
UNet	79.04	92.79	94.06	88.63	93.42
Ours	86.12	95.89	96.44	92.82	96.11

**Figure 11:**
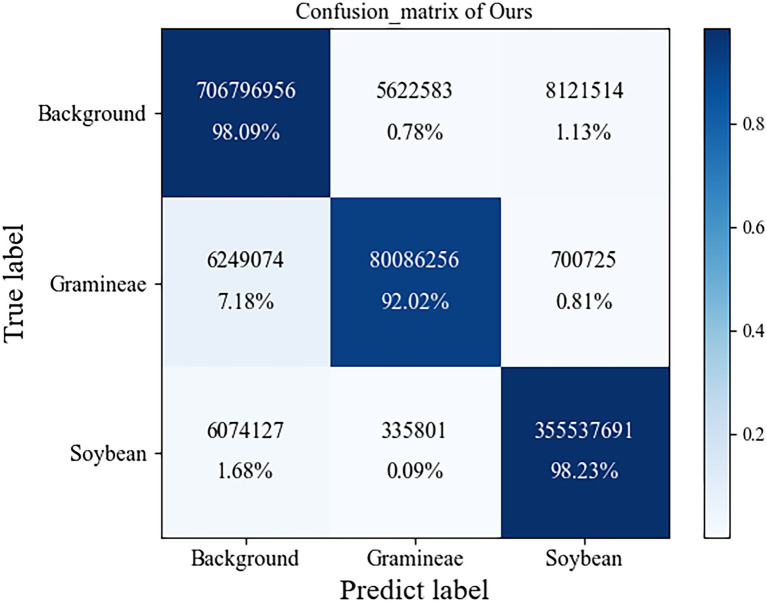
The confusion matrix of our module.

In complex field environments, grass weeds are relatively dense and overlap, making it difficult to distinguish grass weeds. All indicators of the traditional UNet model are better than those of FCN and SegNet because UNet uses skip connections to fuse low-dimensional semantic features extracted in the encoding stage with high-dimensional semantic features to obtain multiscale features and achieve better segmentation results. The model in this paper is superior to other semantic segmentation methods in terms of the intersection ratio, average intersection ratio, and average accuracy. Compared with the traditional UNet model, the average intersection ratio is 2.31 percentage points higher, and the average accuracy rate is 2.69 percentage points higher. Compared with other semantic segmentation methods, it has a significant improvement. The confusion matrix of our module is shown in Figure 11.

**Figure 12:**
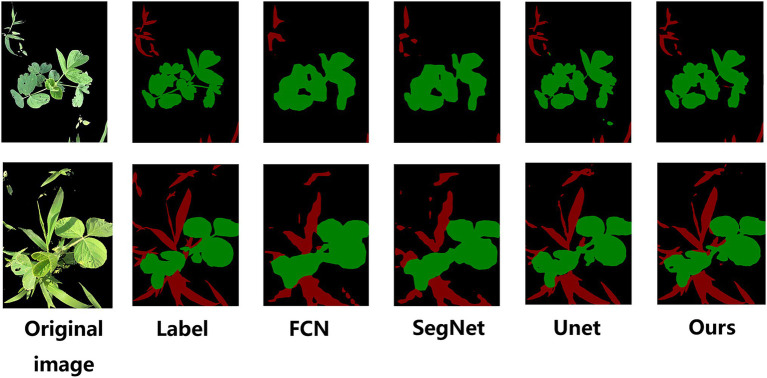
Comparison of the segmentation effects of the different models.

To verify the effectiveness of the method proposed in this paper for weed semantic segmentation, different models are used to test the semantic segmentation results on the same set of images, and the segmentation effect is shown in Figure 12.

Through the segmentation effect map, it is found that the segmentation ability of FCN and SegNet is poor, the edges of the segmented weeds and soybean crops are not smooth enough, and the overlapping parts of the soybean crops and weeds are easily segmented into soybeans. The overall contour segmentation effect of UNet is good, but the details cannot be accurately identified, and misjudgment is prone to occur. The channel attention mechanism SE module and the improved UNet structure designed in this paper can make the model focus more on the detailed segmentation of the overlapping parts of soybeans and weeds, resulting in smoother contours and smoother segmentation renderings of soybeans and weeds with greater detail.

Through the above experiments, the model proposed in this paper is compared with the existing semantic segmentation models in the same experimental environment, which proves that the method in this paper has certain advantages in weed identification.

To evaluate the impact of the various components of the proposed method on the performance of the model, ablation experiments were designed on the soybean weed dataset. After comparing the performance of different models, it is found that UNet has the best segmentation effect. Therefore, UNet is selected as the basic network structure to evaluate the impact of the residual structure with SENet and the improved UNet structure on the performance of the model, as shown in Table 2.

**Table 2:** Performance comparison of the different models.

Model	mIoU%	mPA%
UNet	88.63	93.42
UNet + SE	89.56	94.31
UNet structure improvement	90.32	94.82
UNet + SE + structure improvement	92.82	96.11

Compared with the traditional UNet model, due to the addition of the channel attention mechanism SE module, the weight of each channel is adjusted, the noise and other feature channel weights that are not related to the recognition target are suppressed, and the useful feature weights are enhanced. After adding the channel attention mechanism SE module, the average intersection ratio and average accuracy are improved by 0.93 and 0.89 percentage points, respectively.

To eliminate the semantic gap in the improved UNet, the final feature map used for prediction is obtained by the fusion of upsampling and the feature maps that contain more low-dimensional semantic information in the first five layers. The ratio and average accuracy are improved by 1.69 and 1.40 percentage points, respectively, proving the effectiveness of the structure improvement. After adding the improved UNet structure to the SE module of the channel attention mechanism, the average intersection ratio and average accuracy are improved more significantly; they are increased by 4.19 and 2.69 percentage points, respectively. According to the characteristics of soybean shape and area, appropriate shallow features and deep features are selected for fusion to achieve the expected recognition effect. In summary, the improved UNet and embedded channel attention mechanism SE module are effective.

## Conclusion

To solve the problem of identifying different types of weeds in a natural complex field environment and achieve the goal of spraying different types of herbicides with precise variables to prevent pesticide waste and drug residues, this paper proposes an improved UNet structure and embeds an SE module for soybean weeds in a natural field environment. A grass identification method is applied. Compared with the original UNet model, the overall performance is improved, the low-dimensional semantic features of weeds and soybeans are fully considered in the training samples, appropriate feature layers are selected for fusion and SE modules are embedded to improve the recognition performance; thus, the effectiveness of the method in this paper is proven. The effectiveness of identifying different types of weeds under complex natural conditions in a field provides a valuable reference for subsequent intelligent precision variable spraying and weeding.

## Data Availability Statement

The raw data supporting the conclusions of this article will be made available by the authors, without undue reservation.

## Author Contributions

HY, CB, and HL contributed to the conception and design of the study. ZM preprocessed the data. ZM and HY wrote the first draft of the manuscript. All authors contributed to the article and approved the submitted version.

## Funding

This research was supported by the Science and Technology Development Program of Jilin Province (20190301024NY).

## Conflict of Interest

The authors declare that the research was conducted in the absence of any commercial or financial relationships that could be construed as a potential conflict of interest.

## Publisher’s Note

All claims expressed in this article are solely those of the authors and do not necessarily represent those of their affiliated organizations, or those of the publisher, the editors and the reviewers. Any product that may be evaluated in this article, or claim that may be made by its manufacturer, is not guaranteed or endorsed by the publisher.
